# Plasma Ghrelin Levels Are Associated with Anorexia but Not Cachexia in Patients with NSCLC

**DOI:** 10.3389/fphys.2017.00119

**Published:** 2017-03-01

**Authors:** Susanne Blauwhoff-Buskermolen, Jacqueline A. E. Langius, Annemieke C. Heijboer, Annemarie Becker, Marian A. E. de van der Schueren, Henk M. W. Verheul

**Affiliations:** ^1^Department of Nutrition and Dietetics, Internal Medicine, VU University Medical CenterAmsterdam, Netherlands; ^2^Department of Medical Oncology, VU University Medical CenterAmsterdam, Netherlands; ^3^Faculty of Health, Nutrition and Sport, The Hague University of Applied SciencesThe Hague, Netherlands; ^4^Department of Clinical Chemistry, Endocrine Laboratory, VU University Medical CenterAmsterdam, Netherlands; ^5^Department of Pulmonology, VU University Medical CenterAmsterdam, Netherlands; ^6^Faculty of Health and Social Studies, Department of Nutrition, Sports and Health, HAN University of Applied SciencesNijmegen, Netherlands

**Keywords:** ghrelin, anorexia, cachexia, NSCLC, appetite, weight loss

## Abstract

**Background and Aims:** The ghrelin receptor is one of the new therapeutic targets in the cancer anorexia-cachexia syndrome. Previous studies revealed that plasma ghrelin levels were high in patients with anorexia nervosa and low in obese subjects. We studied to what extent ghrelin levels are related with anorexia and cachexia in patients with cancer.

**Materials and Methods:** Fasted ghrelin levels were determined as well as anorexia and cachexia in patients with stage III/IV non-small cell lung cancer before chemotherapy. Total plasma ghrelin was measured by radioimmunoassay. Anorexia was measured with the FAACT-A/CS questionnaire (cut-off value ≤ 37). Cachexia was determined as >5% weight loss (WL) in 6 months or >2% WL in 6 months in combination with low BMI or low muscle mass. The Kruskal-Wallis test was performed to assess differences in plasma ghrelin levels between four groups: patients with (+) or without (−) anorexia (A) or cachexia (C). Multiple regression analyses were performed to assess differences in plasma ghrelin levels between patients C+ and C− and patients with A+ and A− (adjusted for age and sex).

**Results:** Forty patients with stage III (33%) or stage IV (68%) were recruited, of which 50% was male. Mean age was 59.6 ± 10.3 years. Sixteen patients had no anorexia or cachexia (A−C−), seven patients had both anorexia and cachexia (A+C+), ten patients had anorexia without cachexia (A+C−) and seven patients had cachexia without anorexia (A−C+). The levels of total plasma ghrelin were significantly different between the four groups of patients with or without anorexia or cachexia (*p* = 0.032): the A+C− patients had significantly higher ghrelin levels [median (IQR): 1,754 (1,404–2,142) compared to the A−C+ patients 1,026 (952–1,357), *p* = 0.003]. A+ patients had significantly higher ghrelin levels compared A− patients (C+ and C− combined, β: 304, *p* = 0.020). Plasma ghrelin levels were not significantly different in C+ patients compared to C− patients (A+ and A− combined, β: −99, *p* = 0.450).

**Conclusions:** Patients with anorexia had significantly higher ghrelin levels compared to patients without anorexia. We therefore hypothesize that patients with cancer anorexia might benefit from treatment with a ghrelin receptor agonist to prevent WL and deterioration in physical functioning.

## Introduction

One of the promising new therapeutic targets for cancer-associated weight loss (cachexia) is the ghrelin receptor. Ghrelin is a 28-amino acid peptide that is the natural ligand for the growth hormone secretagogue receptor-1a (Kojima et al., 1999). Ghrelin plays an important role in several physiological processes including increasing appetite by stimulating the production of orexigenic neurons such as neuropeptide Y and agouti-related protein-expressing neuron (Zhang and Garcia, 2015). Ghrelin is produced by endocrine cells of the antrum during periods of fasting (Kojima et al., 1999). Ghrelin levels are high in patients with anorexia nervosa, low postprandial, and low in obesity (Zhang and Garcia, 2015). Ghrelin levels increase in patients who develop anorexia during chemotherapy but remain stable in patients without anorexia during chemotherapy (Shimizu et al., 2003). The evidence on changed ghrelin levels in cancer cachexia however, appears to be inconclusive. Some studies showed increased levels of ghrelin in patients with cachexia compared to patients without cachexia (Shimizu et al., 2003; Karapanagiotou et al., 2009), whereas other studies did not show these differences (D'Onghia et al., 2007; Huang et al., 2007).

Randomized trials with ghrelin and ghrelin agonists have shown promising results regarding improvements in appetite, food intake, lean body mass, and quality of life of patients with cancer cachexia (Garcia et al., 2013; Temel et al., 2016). Still, no significant effect on physical functioning and survival was demonstrated in two recent large randomized double blind phase III trials (Temel et al., 2016).

As cachexia may be accompanied by a loss of appetite (anorexia) and ghrelin is directly involved in the regulation of hunger and appetite, it is hypothesized that the presence or absence of anorexia may intervene in the association between cancer cachexia and ghrelin (Shimizu et al., 2003). To test this hypothesis, we studied associations between plasma ghrelin levels and anorexia and cachexia in patients with advanced NSCLC.

## Materials and methods

In this prospective study, patients with stage III or IV NSCLC and starting with chemotherapy were recruited at the department of Pulmonology of the VU University Medical Center in Amsterdam, The Netherlands.

Exclusion criteria: systemic anticancer treatment in the past month, clinically overt ascites or serious pitting edema, Diabetes Mellitus, current use of high dose of corticosteroids, presence of other active inflammatory disease (for example HIV or active colitis) and insufficient command of the Dutch language. The research protocol was approved by the Medical Ethics Committee of the VU University Medical Center Amsterdam and the study was performed in accordance with the ethical standards laid down in the Declaration of Helsinki of 1975 as revised in 1983. Written informed consent was obtained from all participants.

### Measurements

#### Weight loss and BMI

Body weight was measured (with patients wearing light indoor clothes without shoes) within 0.2 kg on a calibrated scale (Seca type 888). Self-reported body weight 6 months before inclusion was assessed. A correction factor for clothes or clothes and shoes (1.3 kg for females, 1.6 kg for males, 1.6 kg for females, and 2.0 kg for males, respectively) was applied when necessary (Frank and Dunlop, 2000). Relative weight change in 6 months was calculated. Body height was measured using a stadiometer; the patient was standing barefoot and height was determined to the nearest cm. BMI was calculated as the ratio of body weight (kg)/height (m)^2^.

#### SMI

Skeletal muscle area (cm^2^) was measured with SliceOmatic Software V 5.0 (Tomovision, Magog, Canada) using routine CT scans conducted for diagnostic purposes. The fourth thoracic vertebra (T4) was used for the assessment of the skeletal muscle area and in patients without evaluable T4 images, L3 images were used. The structures of muscles were quantified based on pre-established thresholds of Hounsfield Units (HU) (−29 to +150) of skeletal muscle tissue (Mitsiopoulos et al., 1998). Cross-sectional areas (cm^2^) of the sum of all these muscles were computed by summing tissue pixels and multiplying by the pixel surface area for each patient. Skeletal Muscle Index (SMI) was calculated as the ratio of skeletal muscle area (cm^2^)/height (m)^2^.

#### Cachexia

Cachexia was defined as:

– Weight loss >5% in 6 months or
– Weight loss >2% in 6 months in combination with BMI <20 or
– Weight loss >2% in 6 months in combination with low skeletal muscle index (SMI): L3: <55 cm^2^/m^2^ for males, <39 cm^2^/m^2^ for females (Fearon et al., 2011), T4: <66.0 cm^2^/m^2^ for males, <51.9 cm^2^/m^2^ for females (cut-off value p50 for T4, comparable to L3 cut-off values in patients with SMI data on both levels, data not published).

#### Anorexia

– The 12 items of the Anorexia/Cachexia subscale (A/CS) of the Functional Assessment of Anorexia/Cachexia Therapy (FAACT) questionnaire (4th version, Dutch) (Ribaudo et al., 2000) were scored on a five-point Likert scale (0 = not at all, 1 = a little bit, 2 = somewhat, 3 = quite a bit, and 4 = very much). For scoring the FAACT − A/CS, the FACIT manual was applied (Cella, 1997). A lower score indicates less appetite. A decreased appetite was defined as having a score of ≤37 (Blauwhoff-Buskermolen et al., 2016).

#### Ghrelin

Venous blood samples were collected between 7:30 and 9:00 a.m. after an overnight fast. Blood samples were collected using EDTA tubes containing 250 KIU of aprotinin (BD Diagnostics, Plymouth, UK), immediately placed on ice, and centrifuged. Plasma was stored at −80°C until assayed. Total plasma ghrelin was determined by radioimmunoassay (RIA) (EMDMillipore Corporation, Merck Life Sciences, KGaA, Darmstadt, Germany). The intra-assay variation was below 4% and the inter-assay variation was below 5%. The lower limit of quantitation (LOQ) was 240 pg/mL. Analyses were performed at the Endocrine Laboratory of the Department of Clinical Chemistry of the VU University Medical Center.

### Statistical analysis

Statistical analyses were performed using SPSS for Windows v. 23.0 (IBM Corporation, Armonk, NY, USA). Descriptive statistics (count (%) and means ± *SD* or median (IQR), as appropriate) were used to describe the study sample. An independent samples Kruskal-Wallis Test was performed to assess differences in ghrelin levels between four groups: patients with (+) or without (−) anorexia (A) or cachexia (C) because of rather small subgroups. Mann-Whitney *U*-tests were performed to assess the largest difference between subgroups.

Linear regression analyses were performed with logtransformed ghrelin levels to assess differences in ghrelin levels for patients with and without anorexia and with and without cachexia. In multiple regression analyses, adjustments for age and sex were performed. A *p* ≤ 0.05 was considered significant for all analyses.

## Results

Forty patients with stage III (33%) or stage IV (68%) were recruited, of which 50% was male. Mean age was 59.6 ± 10.3 years (Table 1).

**Table 1:** **Patient characteristics (*n* = 40)**.

	***n*** **(%)**
Gender (males)	20 (50)
Age in years^†^	59.6 ± 10.3
Cancer stage	
III	13 (33)
IV	27 (68)
BMI in kg/m^2^^†^	23.9 ± 4.0
FAACT-A/CS^‡^	38 (35–42)

† *Mean ± sd*,

‡ *Median (IQR)*.

Sixteen patients had no anorexia or cachexia (A−C−), seven patients had both anorexia and cachexia (A+C+), ten patients had anorexia without cachexia (A+C−) and seven patients had cachexia without anorexia (A−C+). Of the 14 cachectic patients, 11 patients were diagnosed as such based on >5% weight loss. Two patients were found to be cachectic based on 2–5% weight loss in combination with low SMI and one patient was found to be cachectic based on 2–5% weight loss in combination with low BMI.

**Figure 1:**
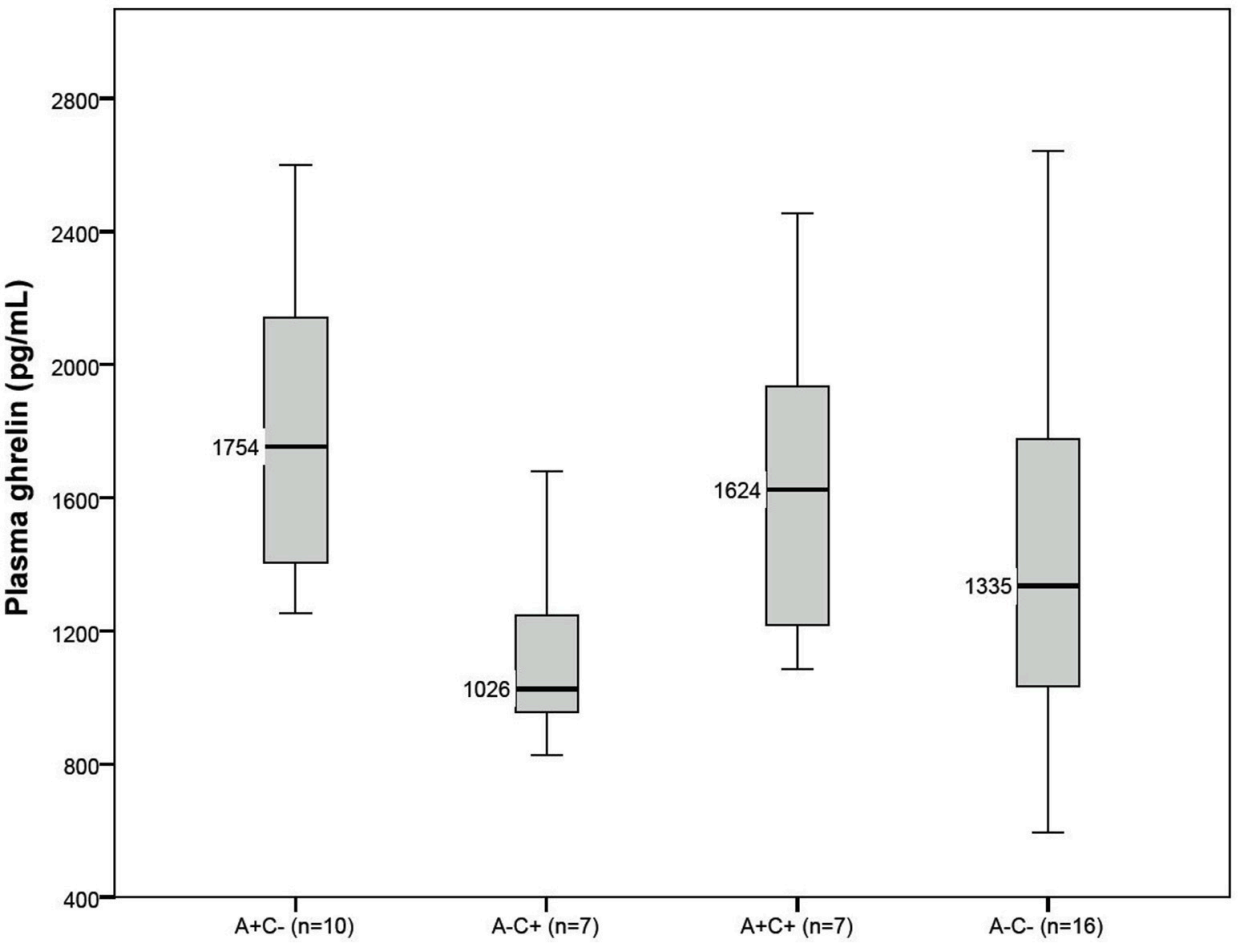
**Boxplots for fasting plasma ghrelin levels for patients with (+) or without (−) anorexia (A) and cachexia (C)**. The thick black horizontal line in each boxplot represents the median value.

Figure 1 shows boxplots with median ghrelin levels for the four groups. Ghrelin levels were significantly different between the four groups (*p* = 0.032). In *post-hoc* analyses, the A+C− patients had significantly higher ghrelin levels (median: 1,754 pg/mL, IQR 1,404–2,142) compared to the A−C+ patients (median: 1,026 pg/mL, IQR 952–1,357, *p* = 0.003).

When cachexia and anorexia were analyzed separately, ghrelin levels were not significantly different between patients with (1,230 pg/mL; IQR 1,026–1,678, *n* = 14) or without cachexia (1,462 pg/mL; IQR 1,212–2,092, *n* = 26; β = −99, *p* = 0.450 adjusted for age and sex). Patients with anorexia (*n* = 17) had significantly higher ghrelin levels (1,624 pg/mL; IQR 1,391–2,102) compared to patients without anorexia (1,212 pg/mL; IQR 957–1,686), *n* = 23; β = 304, *p* = 0.020 adjusted for age and sex).

## Discussion

In NSCLC, we found that patients with anorexia had significantly higher plasma ghrelin levels compared to patients without anorexia. In contrast to previous literature, we did not find associations between ghrelin levels and cachexia.

We hypothesize that anorexia, and accompanying decreased food intake, may lead to increased ghrelin levels in patients with cancer, as endocrine cells of the antrum react to an empty stomach with production of ghrelin (Kojima et al., 1999). This is also supported by the fact that patients with anorexia nervosa also have high ghrelin levels (Zhang and Garcia, 2015).

In patients with cancer, partial resistance to the orexigenic effects of increased ghrelin levels has been hypothesized by Garcia et al. (2005). They compare ghrelin resistance to insulin resistance in type 2 diabetes mellitus, which is overcome by using high doses of insulin. Further elevation in ghrelin levels (three- to four-fold from baseline) may be able to increase appetite and food intake (Garcia et al., 2005). Randomized trials with ghrelin and ghrelin agonists have shown positive results regarding improvements in appetite, food intake, lean body mass and quality of life of patients with cancer cachexia (Garcia et al., 2013; Temel et al., 2016). However, no significant effect on physical functioning and survival was demonstrated in two well-designed large phase III double blind randomized controlled trials (Temel et al., 2016). As anorexia may be present in the pre-cachectic stage and precede significant weight loss and deterioration in physical functioning, future studies should consider treatment with ghrelin in patients with cancer anorexia (for example, in the stage of pre-cachexia) rather than patients with cachexia, in order to study whether significant weight loss and deterioration in physical functioning can be prevented. Literature search resulted in only one small study in seven patients with severe cancer anorexia and this study had promising result: ghrelin infusion resulted in a marked increase in energy intake of 31% and higher meal appreciation scores (Neary et al., 2004). Future studies with larger sample sizes with important end points such as quality of life are of interest, which preferably also include measurement of plasma ghrelin levels in order to learn more about the physiology of ghrelin in patients with cancer cachexia.

In our study we measured only total ghrelin and not acylated (“active”) ghrelin and deacylated (“inactive”) ghrelin separately. The effects of ghrelin on appetite have been assigned to active ghrelin, however the major form of ghrelin in serum is deacylated ghrelin. In a study of Garcia and colleagues, active ghrelin levels and the active to total ghrelin ratio were significantly increased in subjects with cancer-induced cachexia, compared with cancer patients without cachexia and non-cancer controls (Garcia et al., 2005). Nevertheless, there is growing evidence supporting that deacylated ghrelin is also closely linked with food intake and gut motility (Chen et al., 2009). In future studies, acylated and deacylated ghrelin should be analyzed separately in order to learn more about the differences between the two subforms.

We need to remark that absolute reported concentrations of plasma ghrelin are dependent on the analytical method used, therefore we advise to be careful when comparing our reported absolute values with literature on ghrelin levels in humans measured with other methods. Also, when comparing our results to the results of other studies, attention should be paid to the used definitions of anorexia and cachexia. We have used the most recent published classifications of anorexia and cachexia, however this makes comparison to other studies (which used other definitions of anorexia and cachexia) difficult.

This is the first study on plasma ghrelin levels and associations with anorexia and cachexia in patients with cancer. In conclusion, patients with anorexia had significantly higher ghrelin levels compared to patients without anorexia, whereas no differences were found between patients with and without cachexia. In future studies, the effect of ghrelin (agonists) in the treatment of cancer anorexia should be evaluated to prevent severe weight loss and improve clinical outcomes such as physical functioning, quality of life, and survival.

## Author contributions

Conception and design: SB, JL, HV, Md. Collection and assembly of data: SB. Data analysis and interpretation, manuscript writing, and final approval of manuscript: All authors.

## Funding

This study was financially supported by Fonds NutsOhra (1002-039). The funder had no role in study design, data collection, and analysis, decision to publish or preparation of the manuscript.

### Conflict of interest statement

The authors declare that the research was conducted in the absence of any commercial or financial relationships that could be construed as a potential conflict of interest.
